# Virtuell abgehaltene DEGUM-zertifizierte Kurse im Kopf-Hals-Bereich – eine sinnvolle Ergänzung zum konventionellen Kursformat?

**DOI:** 10.1007/s00106-023-01413-8

**Published:** 2024-02-14

**Authors:** Gabriel Hillebrand, Martin Gartmeier, Nora Weiss, Luca Engelmann, Anna Stenzl, Felix Johnson, Benedikt Hofauer

**Affiliations:** 1 Klinik und Poliklinik für Hals‑, Nasen- und Ohrenheilkunde, Klinikum rechts der Isar der TU München, Ismaninger Straße 22, 81675 München, Deutschland; 2 TUM Medical Education Center, Lehrstuhl für Medizindidaktik, medizinische Lehrentwicklung und Bildungsforschung, Fakultät für Medizin, Klinikum rechts der Isar, Nigerstraße 3, 81675 München, Deutschland; 3 Klinik für Hals, Nasen und Ohrenheilkunde, Medizinische Universität Innsbruck, Anichstraße 35, 6020 Innsbruck, Österreich

**Keywords:** Lehre, Ultraschall, Medizinische Fortbildung, Digitale Technologie, COVID-19, Kopf und Hals, Teaching, Ultrasound, Medical education, Digital technology, COVID-19, Head and neck

## Abstract

**Hintergrund:**

Die Ausbildung im klinischen Ultraschall hat eine hohe Relevanz für die Tätigkeit als HNO-Arzt erlangt. Trotz der hohen Nachfrage nach standardisierten und zertifizierten Ausbildungskursen und vielversprechender Daten zu webbasierten und virtuell durchgeführten Ultraschallfortbildungen aus anderen Fachbereichen existieren bisher keine Untersuchungen zur Machbarkeit von rein virtuell durchgeführten, gemäß der Deutschen Gesellschaft für Ultraschall in der Medizin (DEGUM-)zertifizierten Kopf-Hals-Ultraschallkursen.

**Zielsetzung:**

Ziel der Arbeit ist deshalb die qualitative und semiquantitative Analyse der ersten rein virtuell durchgeführten DEGUM-zertifizierten Kopf-Hals-Ultraschallkurse.

**Material und Methoden:**

Im Jahr 2021 wurden 3 rein virtuelle, webbasierte DEGUM-zertifizierte Kopf-Hals-Ultraschallkurse durchgeführt sowie anschließend mittels Fragebogen inklusive Lernkontrolle qualitativ analysiert.

**Ergebnisse:**

Die rein virtuelle Durchführung von DEGUM-Kopf-Hals-Ultraschallkursen erwies sich als machbare Alternative zum konventionellen Kursformat mit einer hohen Akzeptanz unter den Teilnehmern. Die fehlende praktische Übung durch die Teilnehmer bleibt dabei ein relevanter Kritikpunkt.

**Schlussfolgerung:**

Eine zunehmende Verlagerung von Fortbildungsveranstaltungen in den virtuellen Raum scheint auch im Bereich der Lehre sonographischer Fähigkeiten je nach gegebenen Grundvoraussetzungen sinnvoll, jedoch weist eine vollständig virtuelle Durchführung sonographischer Lehrinhalte Defizite in der Übermittlung und Überprüfbarkeit des praktischen Lernerfolgs auf.

## Abstract (en)

**Background:**

Training in clinical ultrasound has become highly relevant for working as an otorhinolaryngologist. While there is a high demand for standardized and certified training courses, until recently, there was no possibility to attend web-based and exclusively virtual head and neck ultrasound courses certified by the *Deutsche Gesellschaft für Ultraschall in der Medizin* (DEGUM; German Society for Ultrasound in Medicine).

**Objective:**

The aim of this study was to provide a qualitative and semi-quantitative analysis of the first purely virtual DEGUM-certified head and neck ultrasound courses.

**Materials and methods:**

In 2021, three purely web-based DEGUM-certified head and neck ultrasound courses were carried out and then qualitatively analyzed using questionnaires including an examination.

**Results:**

The purely virtual implementation of head and neck ultrasound courses proved to be a viable alternative to the conventional course format, with a high level of acceptance among the participants. The lack of practice among the participants remains a relevant criticism.

**Conclusion:**

A more dominant role of web-based and remote ultrasound training is likely and should be considered as an alternative depending on existing conditions. Nevertheless, acquisition of practical sonographic skills remains a major hurdle if courses are purely digital.

## Neue Lehrmethoden

Die Ultraschalluntersuchung hat als nichtinvasives und vielseitiges Bildgebungsverfahren die medizinische Diagnostik und sogar manche Interventionen in der Hals‑, Nasen- und Ohrenheilkunde (HNO) deutlich vereinfacht und verbessert. In den letzten Jahrzehnten hat die sonographische Bildgebung bemerkenswerte Fortschritte in Bezug auf Bildqualität, mobile Anwendbarkeit und Funktionalität durchlaufen. Die Fähigkeit, Ultraschalldiagnostik kompetent anzuwenden und zu interpretieren, ist auch in der HNO zu einer entscheidenden Fähigkeit für Ärztinnen und Ärzte geworden und verbessert die diagnostische Genauigkeit, erleichtert eine angemessene Behandlungsplanung, reduziert den Bedarf an invasiven Eingriffen oder zusätzlichen bildgebenden Untersuchungen und verbessert hierdurch die Patientenversorgung und Ergebnisse [20]. Diese Entwicklungen haben zur Folge, dass die Bedeutung der Lehre und Vermittlung sonographischer Fähigkeiten in der medizinischen Ausbildung gleichsam eine hohe Relevanz erlangt hat. Ultraschallfortbildungen stellen logistische und pädagogische besondere Herausforderungen dar, denn in der (virtuellen) Lehre von sonographischen Fähigkeiten bestehen im Vergleich zu anderen bildgebenden Untersuchungen einige verfahrensbedingte Hürden und Besonderheiten. Zunächst können Ultraschallgeräte und ihre Bedienung insbesondere für Anfänger komplex sein. Der Aufbau der Geräte ist nicht einheitlich und variiert je nach Hersteller, Modell und Generation teils erheblich. Um dem einzelnen Nutzer beizubringen, mit den Geräten effektiv umzugehen und den vollen diagnostischen Mehrwert auszuschöpfen, sind gründliche Kenntnisse und eine geduldige Einweisung erforderlich. Eine weitere Hürde für die Lehrvermittlung der Interpretation von Ultraschall ist die dynamische Natur des Verfahrens. Bilder werden in Echtzeit erzeugt, sodass die Lernenden die Fähigkeit entwickeln müssen, bewegte Bilder zu interpretieren und anatomische Strukturen dynamisch zu identifizieren. Dies erfordert Übung und ein ausgeprägtes Verständnis der Anatomie. Darüber hinaus gelingt die aussagekräftige Darstellung des untersuchten Situs nur mit einem gewissen Grad an eingeübter Hand-Auge-Koordination: Bei der Ultraschallbildgebung muss der Schallkopf am Körper des Patienten optimal gehalten und bewegt werden, um höchste diagnostische Aussagekraft zu erhalten. Zuletzt spielt für den an der Ausbildung in der Ultraschalldiagnostik interessierten Arzt aufgrund seiner Echtzeitanwendung und hohen Untersucherabhängigkeit letztlich auch ein eingeschränkter Zugang zu Patienten einen limitierenden Faktor dar, denn hier ist die Schulung der Fähigkeiten zur Muster- und Bilderkennung mit dem reinen Studieren pathologischer Befunde anhand von Bilddatensätzen sicherlich deutlich weniger sinnvoll als beispielsweise beim Erlernen der Interpretation von computertomographischen Befunden. Eine begrenzte Unterrichtszeit, ein überfüllter Lehrplan und der Bedarf an engagierten Lehrkräften und Ressourcen erfordern für viele Fakultäten Kompromisse auch bei der Ausbildung in der Ultraschalldiagnostik [3]. Eine Erweiterung des Unterrichtsformats in den virtuellen Raum stellt daher gerade bei knappen Ressourcen eine sinnvolle Kompensationsmöglichkeit dar. Zudem bietet virtueller Unterricht weitere Vorteile. Hierzu zählen eine besser an die eigenen Bedürfnisse anpassbare, einfachere und einheitliche Ressourcenzuweisung für die Dozenten sowie die räumliche (und teilweise auch zeitliche) Flexibilität für die Lernenden [1, 3–12]. Viele der zuvor genannten Herausforderungen der Ultraschallweiterbildung sind bei einer rein mit Onlinekonferenztools durchgeführten Lehre dennoch schwer zu adressieren, und der in vielerlei Hinsicht größte Vorteil einer rein virtuell geführten Fortbildung, nämlich die ausbleibende Präsenz vor Ort, bleibt deren größte Schwäche.

Simulationen mittels virtueller Realität sind ein sehr vielversprechender Ansatz, jedoch durch den höheren Ressourcenaufwand aktuell für die meisten Kliniken und Fakultäten nicht ohne Weiteres implementierbar. Eine Diskrepanz zwischen vorhandenen Ressourcen in der Lehre und wachsendem Ausbildungsbedarf in Studium und Weiterbildung führt zu einer zunehmenden Suche nach im Vergleich zum Bedside-Unterricht leichter skalierbaren, aber der Natur des Verfahrens angemessenen neuen Lehrmethoden [1, 3]. Ein vielversprechender Ansatz ist dabei der remote-gestützte Unterricht im Sinne eines Livestream-Kurses. Dabei streamen die Teilnehmer mittels Webanwendung Vorträge und ggf. weitere Unterrichtsinhalte wie Demoultraschalluntersuchungen pathologischer Befunde in Echtzeit auf ihre Endgeräte, jedoch besteht keine Möglichkeit zum eigenständigen Sonographieren. Wissenschaftliche Daten zum Nutzen solcher Kursformate liegen der Lehrforschung bereits seit einigen Jahren vor. So zeigten diese einen teils deutlichen Mehrgewinn für die Lernenden, beispielsweise aufgrund einer hierdurch erst ermöglichten Unterrichtsteilnahme bei fehlender Infrastruktur vor Ort und dabei gleichwertigem Lernerfolg oder als wissensvertiefendes Angebot ergänzend zum herkömmlichen Kursformat [2, 3]. Für rein virtuelle gemäß Deutscher Gesellschaft für Ultraschall in der Medizin (DEGUM-)zertifizierte Kopf-Hals-Ultraschallkurse liegen bisher keine Untersuchungen zu Machbarkeit und Nutzen vor. In dieser Studie sollen daher folgende Fragestellungen beantwortet werden:

- Sind die Teilnehmer solch neuer Kursformate bereit, für Sonographiekurse gänzlich auf Präsenzveranstaltungen zu verzichten?
- Nehmen die Teilnehmer einen virtuellen Sonographiekurs als ausreichend und gleichwertig zu Präsenzkursen wahr?
- Gelingt eine für die Teilnehmer nachvollziehbare Demonstration sonographischer Befunde anhand der Livesonographie im gewählten, rein virtuellen Format?

## Studiendesign und Methoden

### Methodik

In Zusammenhang mit der COVID-Pandemie und den damit verbundenen Einschränkungen auch für Weiterbildungskurse im Rahmen der Universitätsmedizin erfolgten die DEGUM-zertifizierten Ultraschallkurse der Hals‑, Nasen- und Ohrenklinik der Technischen Universität München des Jahres 2021 erstmals webbasiert und ausschließlich online. Hierbei handelt es sich um die erste rein virtuell durchgeführte Fortbildungsveranstaltung für DEGUM-zertifizierte Kopf-Hals-Sonographie. Es wurden 3 virtuelle Ultraschallkurse mit den Modulen Speicheldrüsensonographie, farbkodierte Duplexsonographie und der DEGUM-Abschlusskurs durchgeführt. Das Format bestand wie auch bei den bisherigen Kursen aus Vorträgen, Lernkontrollfragen sowie aus Livesonographie relevanter pathologischer Veränderungen am Patienten durch geschulte Dozenten. Die Livesonographie wurde auf den Bildschirm der Teilnehmer gestreamt. Dabei war es jederzeit möglich, über die Webanwendung den Dozenten Rückmeldung zu geben sowie Fragen zu stellen. Eine Möglichkeit zur eigenhändigen Ultraschalluntersuchung bestand dagegen im neuen Format nicht. Zudem wurden bereits vorab aufgenommene Ultraschalluntersuchungen angeboten, anhand derer strukturiert Befunde erstellt werden sollten. Es erfolgte eine anonymisierte Evaluation der Onlinekurse mit Lernkontrolle mithilfe eines allen Teilnehmern zugesandten Fragebogens mit 19 Fragen zur subjektiven Wahrnehmung der Kurse und 8 Wissensfragen zur Lernkontrolle. Ein positives Votum der Ethikkommission der Technischen Universität München (Studiennummer 2023-122-S-NP) liegt vor. Die Auswertung der gewonnen Daten im Sinne einer qualitativen retrospektiven Studie erfolgte mithilfe eines Tabellenkalkulationsprogramms. Sofern nicht anders benannt, werden die Werte in Mittelwert und Standardabweichung angegeben.

### Stichprobe

Es nahmen 117 ärztliche Kollegen an den 3 webbasierten Ultraschallkursen teil, von ihnen wurden 104 kontaktiert. Davon beantworteten 27 (26 %) den zugesandten Fragebogen (im Folgenden „Teilnehmer“ genannt).

### Demografische Verteilung

Das durchschnittliche Alter der Teilnehmer lag bei 43 Jahren (Standardabweichung 10,8 Jahre), davon 19 Männer und 8 Frauen. Die durchschnittliche Berufserfahrung als klinische Ärzte betrug 13,8 Jahre (Standardabweichung 10,1 Jahre), und Erfahrung mit klinischem Ultraschall bestand im Mittelwert bereits für 10,7 Jahre. Die überwiegende Mehrheit (88,8 %) verwendete dabei im klinischen Alltag den Ultraschall als diagnostisches Tool täglich oder mehrfach pro Woche. Es hatten 6 Teilnehmer (23 %) bereits zusätzlich zu den Web-Kursen zuvor einen DEGUM-Sonographiekurs im Präsenzformat besucht.

## Ergebnisse

### Durchführbarkeit

**Figure 1:**
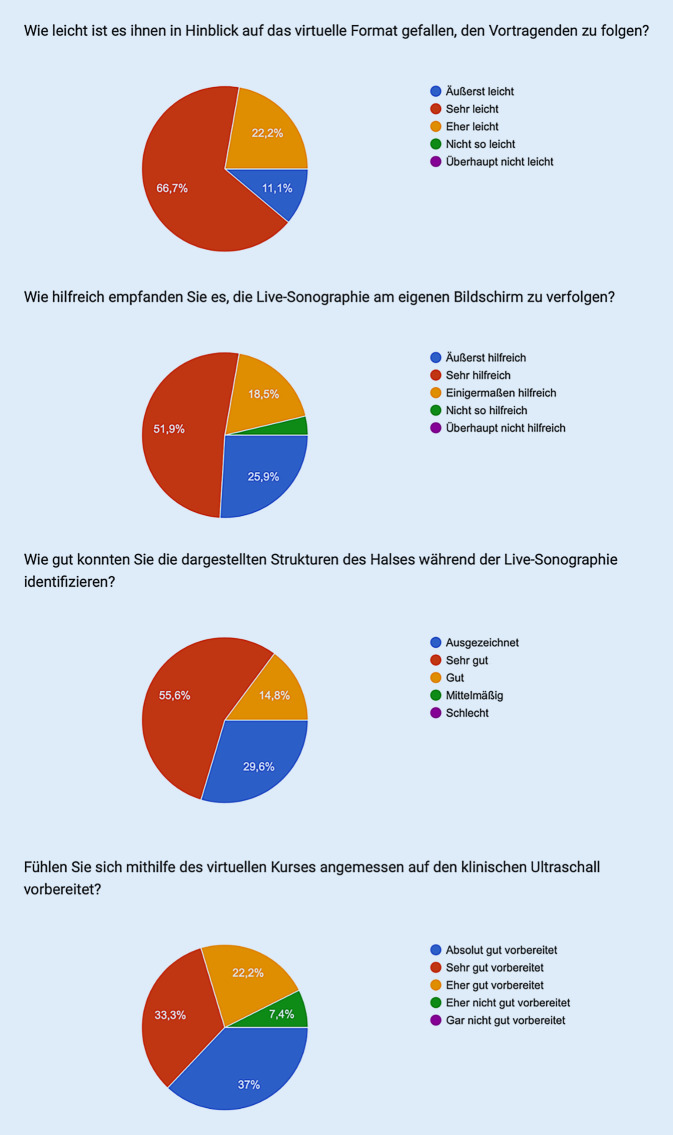

Um den subjektiv erlebten Nutzen und die Schlüssigkeit der Umsetzung der Livesonographie des neuen Fortbildungsformats qualitativ einzuordnen, wurden die Teilnehmer zu einzelnen Aspekten der Livesonographie befragt. Die Live-Übertragung der sonographischen Demonstrationen durch die Dozenten wurde von 26 Teilnehmern (96 %) als hilfreich bewertet, zudem gaben alle 27 Teilnehmer (100 %) an, dass es ihnen im Hinblick auf das virtuelle Format leichtgefallen sei, den Vorträgen zu folgen (Abb. 1). Das Identifizieren der anatomischen Strukturen während der Livesonographie gelang 23 Teilnehmern (85,2 %) „sehr gut“ oder „ausgezeichnet“. Auf den klinischen Ultraschall fühlten sich mithilfe des virtuellen Kurses 19 Teilnehmer (70 %) „sehr gut“ oder „absolut gut“ vorbereitet (Abb. 1). Fazit war, dass eine gute Durchführbarkeit des virtuellen Formats vorlag.

### Akzeptanz

**Figure 2:**
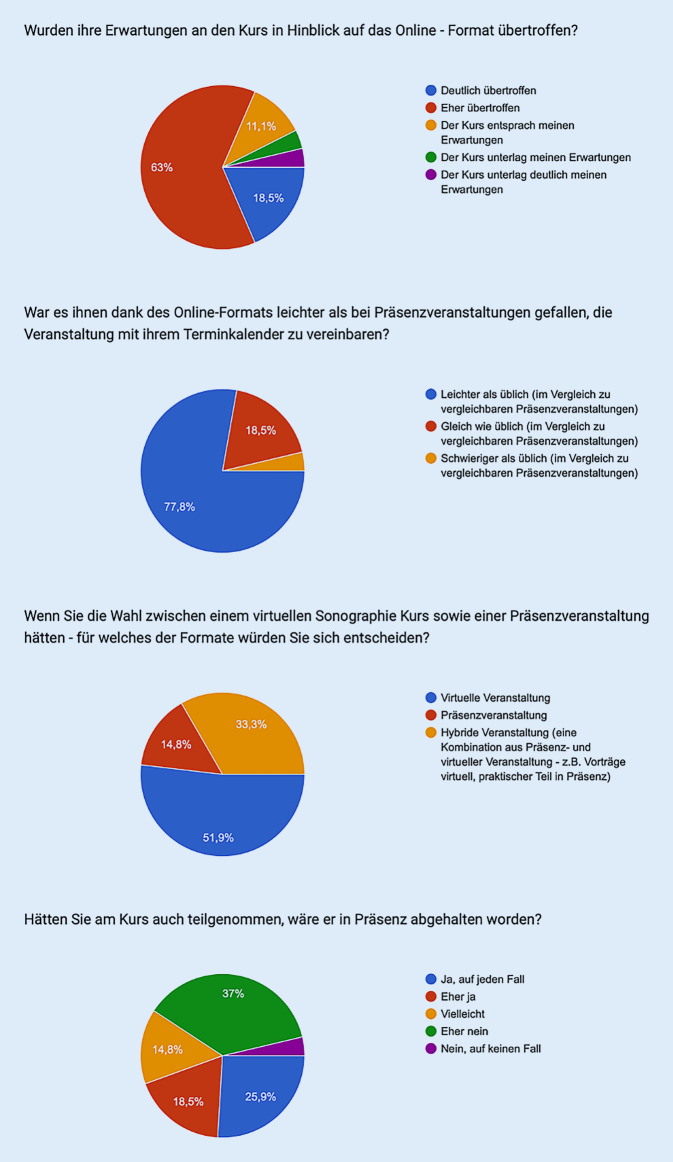

Außerdem sollte mittels des Fragebogens der generelle Zuspruch zum gewählten virtuellen Konzept erfasst werden. Die Erwartungen an das Kursformat wurden dabei aus Sicht einer deutlichen Mehrheit von 22 Teilnehmern (81,5 %) übertroffen (Abb. 2). Bei der Frage nach dem bevorzugten Format, ob virtuell oder in Präsenz, entschied sich eine Mehrheit von 14 Teilnehmern (51,9 %) für eine ausschließlich onlinebasierte Durchführung der Kurse und gegen herkömmliche Kursformate (14,8 %) oder Hybridkurse (33,3 %). Eine Durchführung in Präsenz statt virtuell wäre dabei für 11 der Teilnehmer (40,7 %) eher keine Option gewesen (Abb. 2). Es bestand also hohe Akzeptanz einer rein virtuellen Fortbildung.

### Vorteile

Um die Gründe für die deutliche Bevorzugung virtueller Formate näher einzuordnen, wurden die Teilnehmer nach dem ihrer Ansicht nach größten Vorteil des webbasierten Lernens gefragt. Dieser liegt für die Befragten überwiegend in der hinzugewonnen zeitlichen Effizienz und damit einhergehender besserer terminlicher Vereinbarkeit durch die nicht mehr notwendige Anreise. Die Aufwandsersparnis galt demnach als relevantester Aspekt.

### Nachteile

Zudem wurden die Teilnehmer nach dem ihrer Ansicht nach bestehenden größten Nachteil des Onlineformats befragt. Beinahe einhellig bestand die größte Schwäche aus Teilnehmersicht in der geringeren praktischen Interaktionsmöglichkeit mit den Ultraschallgeräten und der komplett wegfallenden Möglichkeit zur sofortigen Anwendung des zuvor erlernten Wissens aus der Livesonographie und den Vorträgen. Es bestand also keine Möglichkeit zum „Hands-on“.

### Lernerfolg

**Figure 3:**
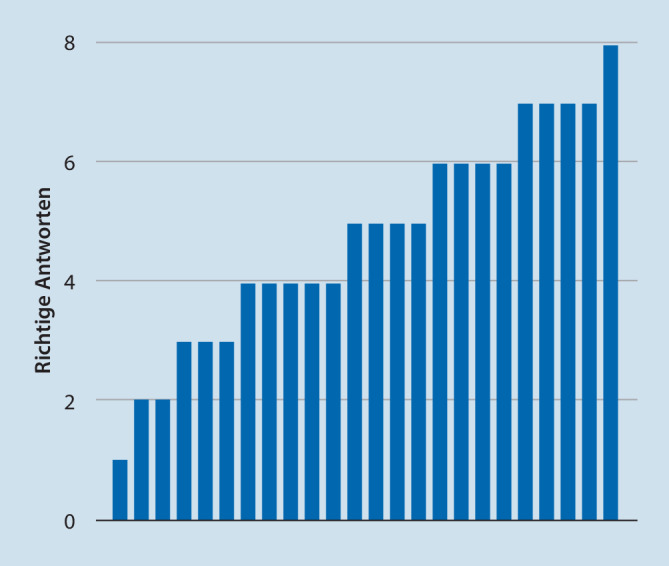

Jeder Teilnehmer beantwortete neben den Fragen zur qualitativen Einschätzung des Kurses 8 Wissensfragen zu Inhalten der Kurse im Multiple-Choice-Format mit 5 Antwortmöglichkeiten und je einer richtigen Antwort. Hierbei wurden durchschnittlich 65 % korrekte Antworten gegeben. Legt man eine Bestehensgrenze von 70 % richtigen Antworten zugrunde, so lagen 37 % der Teilnehmer oberhalb dieser Grenze (Abb. 3). Eine strukturierte Evaluation der praktischen Fähigkeiten erfolgte dabei nicht. Das Abschneiden in der Lernkontrolle lag geringgradig unter den Erwartungen.

## Diskussion

Soweit es den Autoren bekannt ist, erfolgten im Jahr 2021 die ersten rein virtuellen DEGUM-zertifizierten Kopf-Hals-Sonographiekurse. Eine wissenschaftliche Evaluation solcher Kurse lag bisher nicht vor und erfolgte nun erstmalig.

### Zielgruppen

Hierbei verdeutlichen die Ergebnisse, dass eine rein virtuelle Unterrichtsform unter Verzicht auf praktische Übungen eine praktikable Alternative mit hoher Akzeptanz sowie hohem subjektivem Mehrgewinn für die Teilnehmer darstellt, die von den meisten auch für zukünftige Kurse bevorzugt werden würde. Zudem erweist sich die Durchführung solcher Kurse mit Livestreaming der durch die Dozenten durchgeführten Ultraschalluntersuchung als praktikable Alternative zur Demosonographie vor Ort, wobei die Untersuchungsbefunde für die Teilnehmer einfach nachzuvollziehen sind. Die Daten verdeutlichen zudem, dass eine Erweiterung der Zielgruppe durch die webbasierte Durchführung von Ultraschallkursen stattfinden kann. Denn einerseits werden virtuelle Kurse generell den konventionellen bevorzugt, und andererseits kommen zudem Kurse in Präsenz für einen beträchtlichen Teil der Teilnehmer nicht infrage, wobei gemäß der vorliegenden Untersuchung die Anreise trotz prinzipiell gegebener Infrastruktur im deutschsprachigen Raum den maßgeblichen Grund hierfür darstellen könnte.

### Limitationen

Eine möglicherweise bedeutende Limitation dieser Studie liegt im Selektionsbias der Studie. Zum einen kann dieser allein aufgrund der geringen Rücklaufquote von rund 26 % entstehen. In vergleichbaren Studien sind ähnlich niedrige Quoten zwar häufiger anzutreffen, jedoch sind die Fallzahlen meist deutlich höher [3, 21]. Aufgrund der hier zusätzlich gegebenen eher niedrigen Fallzahl kann eine Verzerrung der Ergebnisse nicht ausgeschlossen werden. Dazu hinzukommend geben zudem 40 % der Teilnehmer an, sie hätten nicht am Ultraschallkurs teilgenommen, hätte der Kurs nicht virtuell, sondern unter der Voraussetzung physischer Anwesenheit stattgefunden. Dies lässt sich so interpretieren, dass obwohl die Kurse erstmalig virtuell stattfanden, möglicherweise bei einer relevanten Zahl der Teilnehmer bereits eine positive Voreingenommenheit gegenüber virtuellen Fortbildungskursen im Generellen vorhanden war, da diese in anderen Fachbereichen und bei anderen Themenschwerpunkten nach über einem Jahr pandemiebedingter Einschränkungen bereits gang und gäbe waren. Bisherige wissenschaftliche Erhebungen zu rein webbasierten Unterrichtsformaten in der Sonographie lagen bereits insbesondere für den Bereich der universitären Lehre im Rahmen der medizinischen Curricula vor [19], weniger Untersuchungen bestehen bisher für rein ärztliche Fortbildungen. Häufig wird bei der Übertragbarkeit der Ergebnisse lehrwissenschaftlicher Studien dabei eine demografisch assoziierte Korrelation in der Akzeptanz virtueller Lehrformate postuliert [5]. Dies scheint gemäß den hier erhobenen Daten nicht vorzuliegen, die Akzeptanz ist unabhängig vom Alter der Teilnehmer hoch. Gemäß der vorhandenen Literatur erscheint dabei die prinzipielle Annahme gerechtfertigt, dass rein virtuelle Lehrformate in der Sonographie unter didaktischen Gesichtspunkten vielversprechende Ergänzungen zu konventionellen Formaten darstellen bzw. diese sogar ersetzen können [1, 6, 8, 9, 13–16]. Diese Annahme einer Gleichwertigkeit stützt sich dabei in den zitierten Studien meist auf ein zumindest vergleichbares Abschneiden in Lernkontrollfragen und teilweise strukturierte Evaluationen der praktischen Fähigkeiten im Anschluss an einen virtuellen Kurs. Über den Nutzen solch neuartiger Formate bei der Vermittlung von Fähigkeiten im Bereich der sonographiegesteuerten Intervention liegen dagegen widersprüchliche Resultate vor [17, 18]. Hierbei sollte nicht unerwähnt bleiben, dass die bisher durchgeführten Studien in Bezug auf die untersuchten Gruppen, den Umfang der Intervention und der Erhebungen sowie die im Ultraschall untersuchten Körperregionen sehr heterogen sind, und nur selten liegen kontrollierte randomisierte Bedingungen vor. Einen solchen Nachweis einer Gleichwertigkeit vermag diese Studie ebenfalls nicht unbedingt zu liefern. Denn auch für die hier präsentierte Arbeit gilt als bedeutende Limitation sicherlich, dass eine vergleichende Aussage zu dem mithilfe des Kurses hinzugewonnen Fachwissen nur eingeschränkt möglich ist, da zwar eine Wissensabfrage erfolgte, hierzu jedoch kein Vergleichswert aus einer Kontrollgruppe eines Präsenzkurses hinzugezogen werden konnte. Zudem erfolgte in dieser Studie keine strukturierte Evaluation der praktischen Fähigkeiten im Ultraschall. So bleiben die Ergebnisse auf eine subjektive Einschätzung durch die Teilnehmer beschränkt. In den erwähnten Lernkontrollfragen zum theoretischen Wissen der Ultraschalldiagnostik aus der vorliegenden Untersuchung erreichten zudem nur gut ein Drittel der Teilnehmer einen Wert mit mehr als 70 % richtigen Antworten. Dies lag damit unter den Erwartungen, die man anhand der Erfahrungswerte aus den Lernkontrollen der bisher bei den Autoren in Präsenz erfolgten Kurse haben würde. Für eine DEGUM-Zertifizierung eines Sonographiekurses ist jedoch grundsätzlich eine gewisse Überprüfbarkeit der praktischen Fähigkeiten im Ultraschall wünschenswert, sodass fraglich bleibt, ob außerhalb der pandemischen Notlage eine regelhafte Ausweitung der Zertifizierungen auf rein virtuelle Kurse erwartbar sein kann.

## Ausblick

Aus der vorliegenden Erhebung unter den Kursteilnehmern erscheint es daher eher naheliegend, im Rahmen von Hybridformaten mögliche Vorteile der jeweiligen Formate weitgehend zu kombinieren. Dies könnte durch die Aufteilung in Präsenz- und Onlineteil zu einer höheren Flexibilität bei der Kursplanung und -durchführung sowohl für Lehrende als auch Lernende führen. Ob solche Hybridformate eine ähnlich hohe Zufriedenstellung der Teilnehmer wie reine Onlineformate zu erzielen vermögen, bleibt dabei zukünftigen Untersuchungen vorbehalten.

## Fazit für die Praxis

- Die Durchführung von Ultraschallkursen als Onlinefortbildung stellt zumindest im Rahmen der pandemischen Notlage eine machbare Alternative mit subjektiv erlebtem hohem Mehrwert für die Teilnehmer dar.
- Größter als Nachteil wahrgenommener Aspekt einer rein virtuellen Kursdurchführung ist die ausbleibende praktische Interaktion mit den Geräten.
- Relevantester positiver Aspekt ist die Durchführbarkeit ohne Anreise und damit in höherer zeitlicher Effizienz für die Teilnehmer.
- Es besteht ein hoher Grad an Akzeptanz virtueller Fortbildungskurse auch im Bereich der Kopf-Hals-Sonographie.
- Eine virtuell gestützte Durchführung von Ultraschallkursen erhöht die Reichweite und vergrößert die Zielgruppe der möglichen Teilnehmer.
